# An alternative methodology for the prediction of adherence to anti HIV treatment

**DOI:** 10.1186/1742-6405-6-9

**Published:** 2009-06-01

**Authors:** I Richard Thompson, Penelope Bidgood, Andrea Petróczi, James CW Denholm-Price, Mark D Fielder, The EuResist Network Study Group

**Affiliations:** 1 School of Life Sciences, Kingston University, Penrhyn Road, Kingston-upon-Thames. KT1 2EE, UK; 2 Faculty of Computing, Information Systems and Mathematics, Kingston University, Penrhyn Road, Kingston-upon-Thames. KT1 2EE, UK; 3 EuResist, Via del Commercio, 36 - 00154 Rome - Italy

## Abstract

**Background:**

Successful treatment of HIV-positive patients is fundamental to controlling the progression to AIDS. Causes of treatment failure are either related to drug resistance and/or insufficient drug levels in the blood. Severe side effects, coupled with the intense nature of many regimens, can lead to treatment fatigue and consequently to periodic or permanent non-adherence. Although non-adherence is a recognised problem in HIV treatment, it is still poorly detected in both clinical practice and research and often based on unreliable information such as self-reports, or in a research setting, Medication Events Monitoring System caps or prescription refill rates. To meet the need for having objective information on adherence, we propose a method using viral load and HIV genome sequence data to identify non-adherence amongst patients.

**Presentation of the hypothesis:**

With non-adherence operationally defined as a sharp increase in viral load in the absence of mutation, it is hypothesised that periods of non-adherence can be identified retrospectively based on the observed relationship between changes in viral load and mutation.

**Testing the hypothesis:**

Spikes in the viral load (VL) can be identified from time periods over which VL rises above the undetectable level to a point at which the VL decreases by a threshold amount. The presence of mutations can be established by comparing each sequence to a reference sequence and by comparing sequences in pairs taken sequentially in time, in order to identify changes within the sequences at or around 'treatment change events'. Observed spikes in VL measurements without mutation in the corresponding sequence data then serve as a proxy indicator of non-adherence.

**Implications of the hypothesis:**

It is envisaged that the validation of the hypothesised approach will serve as a first step on the road to clinical practice. The information inferred from clinical data on adherence would be a crucially important feature of treatment prediction tools provided for practitioners to aid daily practice. In addition, distinct characteristics of biological markers routinely used to assess the state of the disease may be identified in the adherent and non-adherent groups. This latter approach would directly help clinicians to differentiate between non-responding and non-adherent patients.

## Background

Whilst the first cases of AIDS were identified in the USA [1,2], and shortly after in Europe [3,4] it is now known that the disease originated from sub-Saharan Africa [5], which currently holds two thirds of the world's 33.2 million people living with HIV. Recent estimates also suggest that Africa has 1.7 million of the global 2.5 million individuals newly infected during 2007 [6]. During 1983, 3,064 people in the US were found to have AIDS, of which a third died [7]; the number infected had risen to 20,745 by 1987 [8]. The rapid spread of HIV over the early pre-treatment years is testament to the aggressive nature of the disease and the importance of using effective drugs to combat this infection. However, a recent analysis showed that life expectancy at age 20 years had increased by over 13 years since the introduction of combination antiretroviral therapy and currently life expectancy of HIV patients at age 20 is up to two thirds that of the general non-HIV population [9].

At present, HIV is treated with a combination of drugs known as highly active anti-retroviral therapy (HAART) or combined anti-retroviral therapy, which works on the principle of using a combination of different classes of drugs to simultaneously impact a range of viral targets. The extent to which patients adhere to their therapy regime is pivotal to treatment success. Although adherence-nonadherence occurs on a continuum, in the case of HIV treatment exceptionally high levels of adherence (> 95%) to HAART are required to suppress viral replication [10].

Treatment change decisions for patients with AIDS who are undergoing HAART are commonly based on clinical treatment history data, ideally including VL, CD4 and genotype information with adherence assumed, based on practical experience or self-declared. Thus designing successful treatment regimes requires an accurate understanding of factors affecting or affected by patients' adherence.

### The importance of adherence

The side effects from HAART drugs affect most patients and can be debilitating. Since HIV causes long-term infection, patients often become fatigued by the constant necessity to take medication and from their severe side effects. Whilst these drugs are highly effective when taken correctly, incorrect use can lead to the development of resistance. Contrastingly, adherence levels which are too low to generate resistance do not decrease or delay the progression to AIDS [11], but a 10% improvement in adherence can lead to a 28% decrease in the risk of developing AIDS [12].

Whilst research has shown that 100% adherence is not necessary for viral suppression, accumulation of drug resistance increases with adherence where patients have incomplete viral suppression [10,11]. It has been observed that adherent patients have longer periods of successful treatment and lower mortality rates than patients who are less adherent [11,13], but patients with lower levels of adherence carry fewer resistant strains [10,11]. This drug dependent adherence/resistance relationship is typically stronger for protease inhibitors (PI) than for other drug classes [11,13,14]. For example within a population of patients on a single-PI therapy, maximum drug resistance occurs at around 80% adherence [14]. In addition, poor adherence in patients with discordant responses (exhibiting a positive response to treatment in VL but not CD4, or *vice versa*) has been associated with increased mortality, as these patients are thought to be less able to produce complete responses to treatment [15].

The literature provides a wealth of possible methodologies to improve patient adherence. Whilst work in Africa has improved adherence through the instigation of workplace clinics [16], work elsewhere has suggested that improving patients knowledge and understanding of their disease and its treatment, either by nursing staff or community-led through pharmacies, has a positive impact on adherence levels. There is evidence to suggest that the impact upon adherence is minimal when this information is supplied by clinicians. This effect may be due to the impact of the patient-'provider' interaction upon a patient's confidence to adhere. A recent pilot study showed that mobile phone reminders were effective in encouraging patients to consistently maintain high levels of adherence throughout the study period [17]. However, the researchers found that adherence waned following the study and that longer monitoring periods may be more beneficial to improving long-term adherence.

Modelling of patient data suggests that patients who feel confident in their ability to adhere to their treatments are more likely to adhere and maintain undetectable VL levels [18]. This notion has been supported by recent patient studies [19]. It has been observed that patients' prognosis is likely to improve [19], through the development of strong patient to care provider relationships and individualised treatment. More recently it has been shown that hospitalisation of patients whilst initialising treatment significantly improves their levels of long-term adherence [20]. Johnson *et al*. suggests that adherence behaviour can be influenced by a minimal educational effort prior to the commencement of retroviral therapy [21] although there is some concern over the long term efficacy when the information content is low.

The ability of patients to understand both their disease and its treatment has a major effect on adherence; by supplying information in a (primarily) pictographic rather than written form, researchers were able to demonstrate improved understanding and compliance by patients with literacy issues [22]. Patients who received didactic interventions were more likely to report high levels of adherence and to achieve undetectable VL level. Self-reporting did not inflate the effect of such interventions, yet objective measures of adherence tend to inflate effect size [23]. For comprehensive reviews of behavioural interventions in HIV treatment and management there are a number of published articles available, including Munro *et al*. [24] and Strathdee & Patterson [25].

### Evidence of adherence or lack of adherence

Given its importance, a plethora of literature focuses on assessment of adherence, typically using medical variables (CD4 counts, VL measures, plasma drug levels, biologic surrogate markers and presence of the antiretroviral medication in the body), inventory-type indicators (pill counts, pharmacy refills, electronic medication monitors), self-reports (patient diaries) or questionnaires on behavior or psychological assessments of factors assumed to predispose the risks for nonadherence [10]. However, a considerable threat to validity is embedded in each of these assessments: In real-life situations and observational studies, medical variables are likely to be confounded by other unmeasured factors (such as co-infection, nonadherence, drug use). The validity and reliability of self-reported information are questionable.

As yet there is no 'gold standard' for adherence measurement in HIV patients and recent research [26] has suggested that the use of multiple measures will be of greater benefit than the continued search for a single defining adherence measure. Current methods can be divided into two forms: i) subjective, such as various self-report and interview based methods and ii) semi-objective, such as the use of MEMS caps and prescription refill rates; however, their link to actual behaviour is dubious.

It is apparent that the use of plasma drug levels to directly measure drug concentrations would be the most accurate and objective way of measuring adherence [27], however using such a method outside the context of a clinical trial has logistical and cost implications, even if patients were to agree to having blood taken regularly for this additional purpose.

Short-term changes in VL (a single low but detectable VL, immediately preceded and followed by undetectable VL measurements) have been shown to be associated with short term decreases in adherence, associated with the development of resistance and/or subsequent treatment failure [28]. In previous work, in order to simplify the analysis it has often been assumed that adherence is a 'constant' state influenced by a short list of factors, and therefore patients were assumed to be either adherent or non-adherent. However Lazo *et al*. [29] recently showed that adherence to treatment is dynamic in nature and the factors affecting increases in adherence are not the same as those affecting decreases in adherence. Recently it has been shown that many patients who miss treatments often do so because they 'simply forgot' [30].

## Presentation of the hypothesis

The importance of having accurate information on adherence is underscored by the fact that in order to achieve successful treatments, physicians must have accurate and reliable information on the effectiveness and efficacy of the prescribed treatment regime. To meet the need for having objective information on adherence, a method is proposed using VL and HIV genome sequence data to observe adherence amongst patients.

Previous work has shown that VL is strongly predicted by patient adherence, whereas drug resistance is a weaker predictor of VL. Bangsberg *et al*. [31] have suggested that the strength of the relationship may vary between clinical settings, drug regimen and even study population. Through modelling factors affecting variation in VL, Llabre *et al*. [32] demonstrated that adherence explains about half of a patient's VL variability. Previously, Moore *et al*. have shown that poor responses to treatments may not be related to the development of drug resistance mutations [15].

Many studies have used current data from patients to establish the relationships between VL and adherence (examples include [15-17,19,22,23,33-37]). It is therefore postulated that the strength of the relationship between adherence and VL is such that where there is no evidence of mutation, levels of adherence can be estimated through patterns of change in VL. The challenge in this approach arises from the need to identify whether the observed changes in VL are due to developed drug resistance or non-adherence. Hence the hypothesis is presented that periods of non-adherence can be retrospectively observed through changes in VL.

It is suggested that the adherence hypothesis could be validated by analysing the rate of change in VL, in the context of observed mutations within the HIV genome. It seems likely that a gradual increase in VL would result from something other than non-adherence, such as low level or intermittent adherence. Non-adherence would be likely to give rise to a sharp increase (high rate of change) in VL, which would appear as 'spikes' in the VL time series. The 'spikes' could then be classified into the 'likelihood of adherence/non-adherence' groupings based upon the rate of change of VL and the presence of mutation.

## Testing the hypothesis

The preliminary work relating to testing this hypothesis requires longitudinal data that includes VL and sequence data from patients. VL points should be collated to produce pairs of VL measurements relating to the start and end points of an increase in log_10 _(VL) of greater than 2 log – In keeping with the clinical practice underlying the Eu*Resist *modelling work, this magnitude of change is suggested as it would give a large enough change to avoid inclusion of insignificant fluctuations in VL [38]. These pairs of time points can then be associated with a HIV sequence collected shortly beforehand, such as within a few months of the first time point in the VL pair.

The HIV pol sequences should be compared to a reference pol sequence and if possible to a previously collected sequence. For information on HIV genomic sequencing that will be used to test the hypothesis, see the background documents of the Eu*Resist *prediction engine [39]. In order to identify a sequence as being mutated it is necessary to identify changes relative to both the reference sequence and a prior sequence, this will ensure that mutations in the sequence are not incorrectly associated with the current treatment. Direct comparison of each sequence with the reference sequence using tools such as BLAST [40] and FASTA [41] would facilitate the removal of mutation sequences occurring outside the time frame of interest in order to identify mutations present within each treatment change episode [38].

**Figure 1:**
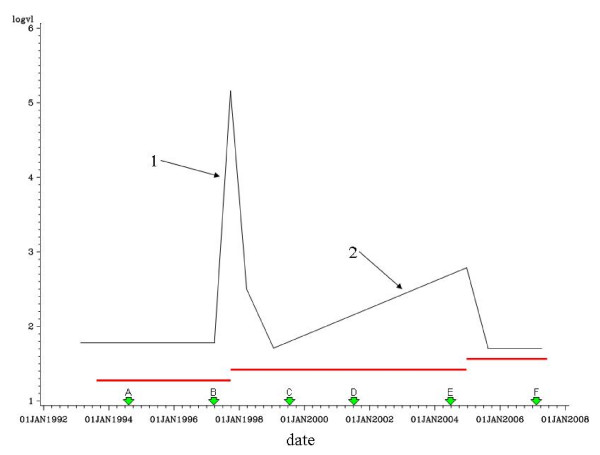
**Representative data for a theoretical patient**. log_10_(viral load) is shown in Black, Treatment periods shown in red; HIV pol gene sequences represented by green arrows.

Once the mutation status has been ascertained, rates of change in VL can be compared to a threshold value in order to decide whether the patient is likely to have been adherent during the period shortly before and during the spike. As an example, Fig 1 shows illustrative data for a hypothetical patient. The mutation status is defined as a change in the sequence relative to the previous sequence and to the reference sequence. For instance, in the figure sequence B would be defined as mutated if it differed from the reference sequence and also from sequence A.

The rate of change in log_10 _(VL) is defined as the increase in log_10_(VL) divided by the time period over which it occurred. For example, for spike 1 the rate would be (5.2–1.8) divided by (number of days between 1 June 1998 and 1 Oct 1998). It is clear from the graph in Fig 1 that slope 2 is too shallow to be considered a spike but if sequence B were found to be unmutated and the slope at spike 1 was to exceed an as yet undetermined threshold value, the possibility that this patient had been non-adherent during the treatment period could be assessed.

## Implications of the hypothesis

Whilst clearly preliminary work to validate this hypothesis needs to be undertaken, if validated the information gained could be used to corroborate and clarify verbal adherence discussions between patients and care-providers, enabling care-providers to recognise adherence problems more accurately and identify opportunities to provide appropriate (re-) education, assistance or regimen change, to minimise disease progression.

If established, this approach will clearly allow for the level of adherence to drug therapy regimes to be monitored. Additionally, such an analysis would facilitate a more accurate assessment of the progression of the patient in terms of their disease status relative to treatment stratagem with a known integrity continuum, resulting in improved treatment and better life expectancy of HIV infected patients.

In clinical practice, removed from research studies utilising various methods to detail adherence in patients, clinicians routinely rely on verbal discussions to identify periods of non-adherence. With a clear expectation for adherence, it is plausible that some (or many) patients are unwilling to openly admit non-adherence, leading to inadvertently misleading their treating physicians. The utilisation of VL data, with knowledge of the presence (or lack) of drug resistant mutations, should allow clinicians to form a more accurate picture of the adherence levels of their patients. This will allow treatment change events and clinical management alterations to be made that may prevent or reduce the occurrence of patient non-adherence to the therapeutic regime. It should be noted however, that the levels of accuracy in this type of analysis are likely to be dependent on the time periods between VL measurements, which are typically every six months in patients with HIV. The limitations of this hypothesis as applied to clinical practice include the access to and costs of sequencing.

Therefore, it is envisaged that the hypothesised approach will serve as a first step on the road to clinical practice. The information on adherence inferred from clinical data is a crucially important feature of treatment prediction models provided for practitioners to aid daily practice. In addition, distinct characteristics of biological markers routinely used to assess the state of the disease may be identified in the adherent and non-adherent groups. This latter approach would directly help clinicians to differentiate between non-responding and non-adherent patients.

## Competing interests

The authors declare that they have no competing interests.

## Authors' contributions

All authors have contributed to the hypothesis development, drafting and critically reviewing the manuscript. Eu*Resist*: the consortium supplied the databases upon which the hypothesis was formulated. All authors have read and approved the final manuscript.

## Authors' informations

IRT is a PhD student, PB is a Principal Lecturer in Statistics, JDP is a Principal Lecturer in Computing, AP is a Reader in Public Health, MDF is a Reader in Medical Microbiology. Eu*Resist *is a research consortium funded under the EC's FP6 IST programme (for member institutions and researchers, see ).
